# Safe acceptance in the nurses’ cultural care of medical tourists in Iran: a qualitative study

**DOI:** 10.1186/s12913-023-09378-8

**Published:** 2023-04-25

**Authors:** Hero Hamzehpour, Tahereh Ashktorab, Maryam Esmaeili

**Affiliations:** 1 Department of Nursing, Faculty of Nursing and Midwifery, Tehran Medical Sciences Islamic Azad University, Tehran, Iran; 2 Department of Management, Faculty of Nursing and Midwifery, Tehran Medical Sciences, Islamic Azad University, Tehran, 00 98 912 236 1149 Iran; 3 Nursing and Midwifery Care Research Center, Critical Care Department, School of Nursing and Midwifery, Tehran University of Medical Sciences, Tehran, Iran

**Keywords:** Qualitative research, Medical tourism, Nursing care, Culture, Safe acceptance

## Abstract

**Background:**

Medical tourism is traveling to another country to promote, restore and maintain health, recreation, and pleasure. There are different types of health tourism, including medical tourism, recovery tourism, and preventive tourism. This study aimed to explain safe acceptance in the nurses’ cultural care of medical tourists in Iran.

**Methods:**

In this qualitative study, 18 semi-structured interviews were conducted with nurses, patients, and patients’ relatives, who had been selected by purposeful sampling in 2021–2022. The interviews were recorded, transcribed, and then analyzed by conventional content analysis.

**Results:**

The statistical analysis revealed that the main theme of this study was safe acceptance which included the five categories of trust building, safety, maintaining comfort and peace, stress control, and identifying patients’ expectations.

**Conclusions:**

The present study demonstrated that safe acceptance of cultural care was essential to medical tourism. Iranian nurses were aware of the factors that affected cultural care and the safe acceptance of medical tourists. Moreover, they carried out the necessary measures to achieve safe acceptance. In this regard, solutions such as developing a comprehensive and mandatory national qualification program and evaluating its periodic performance in this field are suggested.

## Background

Medical tourism is a new concept in modern medicine and refers to patients who travel abroad for medical treatment [1, 2]. Medical tourists have a different cultural and social background than their care providers [3, 4]. Cultural competence and intercultural sensitivity are critical in health tourism. The ability of healthcare providers to put foreign clients at ease is a momentous factor in communication, clinical outcomes, and the reputation of healthcare providers [5]. “Culture” has been mentioned as a determining factor in shaping the patterns of health tourism and is considered the most significant patient-centered element of health care [3, 4]. Providing “culturally appropriate” services to patients will lead to effective interaction between patients and healthcare providers, increasing satisfaction, trust, and healthcare quality [3, 6, 7].

With international health care rising, health and medical tourism concepts, intercultural competence, and cultural safety have become more influential. In the healthcare section, the healthcare providers’ ability to provide culturally appropriate services depends on the “cultural competence” of healthcare workers [8]. Lack of this ability can have adverse consequences, such as the risk of misdiagnosis due to miscommunication between medical staff and patients. Cultural-centered interaction enables nurses to discover a new perspective while helping patients [9].

Unsafe cultural practice is any practice that demeans or undermines the patients’ cultural identity or their families. It can include religious symbols, individual behaviors (eye contact and gender insensitivity), or social actions such as observing or not observing certain rituals. The unsafe cultural practice comprises any action that diminishes, demeans, or disempowers an individual’s cultural identity and well-being [10, 11]. Establishing trust is one of the critical issues in effective interaction between patients and healthcare providers. In this regard, Rasiah et al. (2020) stated that trust is often associated with effective communication and interaction. It also facilitates patients’ information disclosure and enables caregivers to make the necessary behavioral changes to have more independence in making treatment decisions. Comprehending these issues that affect individuals’ trust in a healthcare provider will assist in designing appropriate operational policies in the healthcare section. Moreover, it affects the practices and behaviors of healthcare providers. This knowledge transfer will make a kind of health care that corresponds to the patient’s requirements [12]. One of the important factors in cultural care was to create a sense of safety. Some studies illustrated that patients experienced a sense of safety when nurses interacted with them and provided appropriate and compassionate services [13, 14].

Medical tourism is a unique type of service influenced by cultural factors. As culture plays a central role in the design of healthcare practice and behavior, it seems that studying the related aspects of culture-centered care is effective in developing a model with a competitive advantage. The quality of services will improve by reducing the stress of foreign patients who are very vulnerable due to their health conditions and have to face additional pressure to tolerate cultural differences [15–17]. The World Tourism Organization (WTO) predicts that revenue from health tourism will reach more than $2 trillion by 2020. According to reports, about 0.5% of the world’s medical tourism income belongs to Iran [18, 19].

Considering that there was no conducted study regarding the safe acceptance of nurses’ cultural care of medical tourists in Iran. Therefore, we aimed to know: “How is cultural care practiced by nurses?“, and “How is it possible to perform safe acceptance of foreign patients?“ Generally, regarding the existing knowledge gap in this field, and since qualitative studies provide a vaster view of the topic under investigation by deeply exploring the experiences of participants, this research was designed with a qualitative approach to explaining the safe acceptance in nurses’ cultural care of health tourists in Iran. This study could lead to a deeper understanding of clinical factors in medical tourism and help organizations involved in improving their services by focusing on the capabilities of healthcare human resources.

## Methods

### Design/ participant

This qualitative study was conducted with the conventional content analysis approach. Data were collected by interviewing 14 nurses; one physician; two patients, and one patient relative, who had been selected by purposeful sampling in the teaching hospitals of Tehran (the capital city of Iran) and Urmia (West of Iran) Medical Sciences Universities. Tehran (capital of Iran) is a touristic city and the first entry point for many medical tourists to Iran. There are many private hospitals and clinics in Tehran, which contain suitable facilities for medical tourists. This city has the best hotels, tourist attractions, free entertainment, and sceneries, and offers excellent health tourism services to tourists with magnificent security. Urmia is in a province with a common border with three countries: Turkey, Azerbaijan, and Iraq. Considering the unique border capacity and the development of health indicators, it is a suitable destination for medical tourists who need health services. Inclusion criteria for nurses were; being a clinical nurse with experience in caring for medical tourists and being willing to participate in the study. Also, to better understand the concept of cultural care, interviews were conducted with a diverse range of participants (patients, patient’s relatives, and physicians). The present study was approved by the Ethics Committee of Tehran Islamic Azad Medical University (IR.IAU.TMU.REC.1399.575) and following the 1964 Helsinki Declaration and standards. The research was anonymous, and all participants were given information about the study and asked for written informed consent to participate.

### Data collection

Data were collected through semi-structured interviews with participants. The interviews were conducted in 2021–2022 by the first author at the participant’s workplace or any other places agreed upon by the participants and in four stages [20]. The interviews lasted between 20 and 60 min (Table 1).

**Table 1:** The demographic characteristics of people who participated in the study

Demographic characteristics	Frequency (%)
Age (Mean ± SD)	35.66 ± 7.37
Sex	
Male	9 (50)
Female	9 (50)
Marital status	
Married	11 (61.11)
Single	7 (38.88)
Educational status	
Bachelor’s degree	12 (66.66)
Master’s degree	3 (1.66)
Doctoral degrees	1 (5.55)
Other	2 (11.11)
Duration of the interview (Mean ± SD)	40.05 ± 12.29
Work experience in the department of international patients in nurses (Mean ± SD)	4.83 ± 2.54

SD: Standard Deviation

The first stage (orientation phase): In this stage, the researcher introduced himself and spoke about the study title, general and specific objectives, and the possible time of the interview. The researcher also requested permitting to record the interview and return it to the participant if needed. The second stage (main question): In this phase, the investigator asked the study’s main question to the participant (How is cultural care of medical tourists practiced by nurses?).

Third stage (probing questions): In this phase, probing questions were also asked of the participants to better explore their experiences and clarify any ambiguities. Examples of Probing questions from nurses included: “In your opinion, what are the cultural differences of medical tourists?”, “How do you consider culture in health care?”, and “How do you provide safety for patients?” An example of exploratory Probing from patients included: “Can you tell us about your experience coming to Iran?”, “What did you expect from Iranian nurses when you came to Iran?”, “What things were important to you as a patient?”, An example of probing questions from the patient’s companion included: “What is the difference between cultural care in Iran and your country?”, “What did you expect from Iranian nurses?”, “What things were important to you as a patient companion”? Fourth stage (ending question): At this stage, the participants were asked to talk about anything else they would want to add or express. The interviews ended when data saturation was reached.

Third stage (probing questions): During this phase, according to the expressed experiences of the participants, they were asked probing questions to well-reviewed their experiences and clarify any ambiguities. The probing questions examples of nurses included: “In your opinion, what are the cultural differences of medical tourists?”, “How do you consider culture in health care?”, and “How do you provide patient safety?” An example of exploratory Probing from patients included: “Can you tell us about your experience coming to Iran?”, “What did you expect from Iranian nurses when you came to Iran?”, “What things were important to you as a patient?”, An example of probing questions from the patient’s companion included: “What is the difference between cultural care in Iran and your country?”, “What did you expect from Iranian nurses?”, “What things were important to you as a patient companion”? Fourth stage (ending question): In this step, participants were asked to talk about anything else they wanted to add or express. The interviews ended when the data was sufficiently saturated.

### Data analysis

The interview was immediately transcribed verbatim after each session and read several times to acquire an understanding of the entire interview. Transcribed interviews were analyzed based on conventional content analysis using framework analysis. Conventional content analysis is an inductive qualitative research method in which you develop codes, categories, and themes from textual data rather than pre-existing theories. Regarding why this method was chosen, it should be commented that the nature of safe acceptance in the cultural care of nurses from medical tourism is a concept that requires its analysis and the relations between its elements and revealing the hidden patterns between them. Also, due to the lack of existing studies on this particular group in Iran and the lack of a defining theory for the researcher, the conventional content analysis approach was used [21]. The interviews’ texts were analyzed by the conventional content analysis method in several steps; which consisted of typing down interview text immediately after each interview, reading the full text of the interview to achieve a general understanding, determining semantic units and primary codes, classifying similar codes into more comprehensive categories, and determining the hidden meaning in the data [22]. The extracted codes were classified and subcategories, using MAXQDATA-2020 software.

### Rigor and trustworthiness

Lincoln and Guba’s criteria were used for rigor. To assess the data’s trustworthiness, the criteria of credibility, confirmability, dependability, and transferability were used. The credibility of data was ensured through long-term engagement with the participants, constant data comparison, member check, and peer check. Bracketing was used to ensure data conformability. Dependability was assessed by asking two members of the research team to audit the study process and findings. Finally, for transferability, a rich analytical description of the study process, methodology, and limitations was provided. The principle of maximum variation was also considered during the sampling [23]. The first author of the article and the research team tried to follow these criteria in the process of data collection.

Lincoln and Guba’s stringent criteria known as credibility, dependability, confirmability, and transferability were used to establish the data’s trustworthiness. The credibility of data was ensured through long-term engagement with the participants, constant data comparison, member check, and peer check. Bracketing was used to ensure data conformability. Dependability was assessed by asking two research team members to audit the study process and findings. Finally, a detailed analytical description of the study process, methodology, and limitations was provided for transferability. The principle of maximum variation was also considered during the sampling [23]. The article’s first author and the research team attempted to follow these criteria in the process of data collection.

## Results

Participants in this study were selected based on acquiring maximum diversity. In the 18 interviews, 758 codes were identified. The coding process was performed as a continuous comparative analysis after removing duplicate codes and merging similar codes. Consequently, the main theme of “safe acceptance” and five categories of “trust building”, “safety”, “Comforting Communication”, “stress control” and " Responding to inconsistent expectations " resulted from data analysis. The participants’ demographic characteristics are demonstrated in Table 2.

**Table 2:** Main and sub‑categories extracted from the data

Theme	category	Some of the related codes
Safe acceptance	Trust building	• Reassurance of patients by considering their culture • Trust building through clarification • The importance of mutual trust
	Safety	• Providing safety in an insecure environment • The importance of the first encounter between the patient and the nurse • The same language creates a sense of safety in patients
	Comforting Communication	• Creating peace with a nurse who speaks the same language • Patient comfort through proper communication • Comforting of patients after explaining the procedures by nurses • The importance of keeping calm upon arrival.
	Stress control	• The importance of reducing patients’ stress upon arrival • Reducing stress through the same language • Eliminating stress by considering patients’ culture
	Responding to inconsistent expectations	• High expectations of patients from nurses • Complaining about nurses for not being at the bedside at the same moment • Expecting nurses to obey the patient because of money

### Safe acceptance

According to the participants, patients with various cultures travel to Iran for medical treatment. These patients are concerned about their first encounter with nurses in the hospital. In this situation, nurses’ behavior with patients creates a feeling of safe acceptance in patients. Safe acceptance is an inner feeling that is constructed following two-way trust between nurses and patients. This is the sense of security and safety, which reduces patients’ stress and provide conveniences. When patients trust the nurses and a two-way trust is formed, they feel safe, their stress reduces, and their expectations are more easily identified. In this situation, safe acceptance will occur.

### Trust building

Trust building is one of the crucial categories in safe acceptance. In their statements, nurses referred to the importance of building trust with medical tourists. They believed that safe acceptance occurs when mutual trust and respect are formed between the nurses and the patients. Participant number 15, a 32-year-old nurse, married, stated that:

*“Mutual trust between the treatment staff and patients is very important in providing care.“ (p14)*.

The nurses stated that one of the ways to build trust between them and their patients is proper communication in the first meeting with the patients. According to the nurses’ statements, patients are stressed on arrival, which means they do not feel safe and secure. When proper communication is formed in the first encounter, the patients will feel relaxed and begin to trust nurses. All these factors create a sense of safe acceptance in patients. Patients also stated that they wait for someone to talk to upon their arrival at the hospital, and trust is formed when nurses talk to them. In this context, a male participant with 30 years of nursing experience stated:

*“At the first stage, appropriate treatment and proper communication are very important, because these things create a sense of security and peace in patients and also make patients trust us.“ (p13)*.

On the other hand, when trust is formed between patients and nurses and safe acceptance is occurred, adherence to treatment increases in patients, they express their wishes more easily and they are less confused. In this context, a 27-year-old nurse, single, with five years of work experience in the international patient department and the intensive care unit stated:

*“If patients trust us and believe in what we do, and also if we treat them well, they will naturally adhere to the treatment more easily, take the prescribed medicines better, and follow our orders.“ (p14)*.

Nurses and patients believed that, first of all, patients should be accepted safely. Nurses also argued that building trust is one of the ways to convey a sense of safe acceptance in patients, which promotes cultural care.

### Safety

The statements of most participants indicated that most medical tourists who go to a foreign country for medical care experience a sense of insecurity, which undermines their peace. Nurses take adequate and effective measures to ensure the patients’ safety. According to nurses and patients, the first need and desire of the patients are to have a translator who speaks their language, making them feel safe and secure. Most participants referred to non-verbal communication as one of the ways to create a sense of safety in patients. In this regard, participant number 17, an international patient who came to Tehran for eye surgery from Kurdistan, Iraq, stated that:

*“At the first encounter when entering into the ward, seeing the facial expression of the nurses and their appropriate behavior gives us peace of mind and safety.“* (p17).

Introducing the ward and providing a private room for patients is a significant factor that facilitates the safe acceptance of patients. The patients stated that when nurses adequately explain about the ward and give patients a private room with privacy, they began to feel safe. The participants stated that providing a private room is a kind of respect for the patients’ culture to create a sense of safety in them. By respecting the patients’ privacy and creating a sense of safety, the nurses ensure the safe acceptance of cultural care. The participant, a 32-year-old woman with one year of experience in the international patient department, states that:

*“Patients who visit us have no problems in terms of admission. By providing a private room, a sense of satisfaction and safety was created in international patients.*“ (p6).

In general, it can be said that safe acceptance is achieved through cultural care by choosing nurses with more professional behavior and the ability to communicate effectively.

### Comforting Communication

Another category that is very important in the safe acceptance of medical tourists is Comforting Communication. At first, Medical tourists do not have enough comfort and peace for some reason. Not knowing the language is one of the reasons why patients lose their peace of mind. Improper communication with the treatment staff and a lack of knowledge about the treatment process would cause patients to lose control and become aggressive and restless. Considering these barriers and creating comforting communication is a way to make safe acceptance. According to the statements of participant number 18, who was one of the patient’s companions and came from Iraqi Kurdistan for the treatment of their child, she stated the following in the context of comforting communication in Tehran and Urmia:

*“I spoke Kurdish and they didn’t understand. I spoke Arabic and they didn’t understand, I spoke English and they didn’t understand. I was very frustrated. I’m not that kind of person, but I sat on the floor crying and screaming in the middle of the ward.“(Urmia) (p18)*.

Patients’ reception by the nurses at the arrival moment, was an effective factor in the patients’ calm. In this regard, one of the patient’s companions said:

*“When we entered the hospital, someone came from the hospital. When that person came, I felt very relieved” (p18). (Tehran)*

To maintain comforting communication with patients and achieve safe acceptance, the nurses take measures, such as checking patients regularly to encourage them, talking to patients even if they are unfamiliar with their language, smiling at patients, explaining the procedures, etc. According to the patients’ and nurses’ statements, selecting a nurse with the same language as the patient is one of the most significant categories in establishing effective communication and maintaining the peace and convenience of the patients. In this regard, participant No. 2, who has 10 years of work experience in the international patient department, also owns a health tourism company in Urmia, stated that:

*“If there is an important task, I ask my colleagues to do it so that I can go and admit the foreign patients, and when they see that I am a Kurdish person and fluent in the Kurdish language, they begin to relax, and their peace of mind increases.“ (p2)*.

Getting to know people’s culture and paying attention to their comfort and peace is one of the most important measures in the safe acceptance of international patients. Where people’s comfort and peace are taken seriously and efforts are made to respect their culture, safe acceptance occurs.

### Stress control

Many patients have a high level of stress on their arrival at the hospital, and according to the participants, many factors have caused their stress. For instance, patients’ admission during the Covid-19 pandemic was one of the Categories that created a high level of stress in patients. Lack of familiarity with the language and inappropriate communication of nurses are also causing additional stress in patients. Moreover, a lack of familiarity with the environment and unfamiliar with laws in Iran are creating confusion and stress in patients. According to the statements of participant number 18, who was one of the patient’s companions and came from Iraqi Kurdistan for the treatment of their child, she stated the following in the context of Stress:

*“When I arrived in Urmia, I was very worried and stressed. My husband knew Farsi, but I didn’t.“ (p18)*.

To reduce the stress of medical tourists, the nurses communicated with the patients and talked to them even when they did not know the patient’s language, but according to the nurses and patients, speaking to the patient even in a language that differs from patients’ language reduces their stress. Appropriate behavior of healthcare workers, explaining medical procedures, and selecting nurses who know the language of patients were among other actions taken by the nurses in this study. For example, participant number 11, a 27-year-old nurse, single, with 5 years of work experience in the international patient department and Intensive Care Unit, stated that:

*“We explained everything to them step by step, and they said that when you explain to us, our stress is reduced.“ (p11)*.

Strange country, not knowing the language, inappropriate communication, unfamiliar environment, and the resulting stress lead to insecurity among medical tourists.

### Responding to inconsistent expectations

Most participants stated that medical tourists have different levels of expectations. The nurses asserted that most international patients have higher levels of expectations than Iranian patients for different reasons, so identifying patients’ expectations and meeting their needs is very important in the safe acceptance of patients. In this regard, a male participant with 30 years of nursing experience stated:

*“Patients’ expectations are only about treatment and care. I mean they expect the services to be of high quality and to be done as soon as possible.“ (p13)*.

Among patients’ expectations, we can refer to their insistence on keeping a pet in the hospital, the expectation of speeding up and facilitating medical services, providing care as soon as possible, etc. Safe acceptance of patients occurs when their expectations are determined and steps are taken to satisfy them. In this respect, participant number 12, who had 15 years of work experience and three years of work experience at the international patient department, stated:

*“Because these patients pay more money than Iranian patients, they think they can have any expectations. They expected to bring dogs and cats to their room. Whatever we said, this is a hospital. You got a private room to be comfortable, but you cannot keep animals in the hospital” (p12).*

## Discussion

The present study aimed to explain the safe acceptance in the nurses’ cultural care of medical tourists in Iran. From the participant’s point of view, the safe acceptance of foreign patients can be classified into 5 categories of “trust building”, “safety”, " Comforting Communication”, “stress control” and " Responding to inconsistent expectations “.

The first category identified in this study was trust building. Trust is essential for effective interpersonal relationships and social life. Patients’ trust in healthcare providers is at the core of healthcare services, which improves patients’ health and well-being [24]. In this study, participants stated that nurses’ trust enables patients to better understand nursing education. Also, trust construction makes patients feel safe and accepted. So that, they ask fewer questions from the nurses and adherence to treatment. Considering the cultural differences of foreign patients also creates trust between nurses and patients. A participant in this regard stated that trust is built when medical personnel respect patients’ cultures and religious beliefs. From the perspective of religious sources, observance of professional ethics, the sanctity of purpose, accountability, use of hopeful words, accuracy in examinations, and listening to patients are the most important trust-building categories [24]. Trust in healthcare providers is a key predictor of patients’ participation in medical care. The formation of trust in patients increases their desire to follow treatment recommendations and have trust in the caregivers’ judgments. It also allows caregivers to make decisions on behalf of patients [25]. Iran-Manesh et al. (2018) examined trust in Muslim medical tourists and found that trust affects the attitude of Muslim medical tourists. A positive attitude creates a sense of satisfaction in medical tourists. Meanwhile, age and level of education affect the adjustment of people’s trust and attitude [26]. Lee et al. (2019) found that the key issues that are effective in trust formation in patients included; committing to meet the patient’s needs, creating an appropriate response system, developing communication skills, and involving patients in all stages of care [27]. Finally, it concluded that trust is a magnificent element in interaction and communication between patients and healthcare providers (physicians, nurses, physiotherapists/occupational therapists). Trust in healthcare providers affects patient management outcomes, particularly in long-term illnesses treatment. It also affects health promotion and prevention outcomes. A trusting relationship and communication between the healthcare provider and patient can have a direct therapeutic effect [12].

Another category found in this study was “safety”. Safety has a Latin root, which means “not to have fear and worry”. Therefore, the literal meaning of safety is “freedom from danger, threat, damage, anxiety, panic, fear, and worry or existence of peace, assurance, comfort, trust and safety”. Maslow asserted that each of the emotional, cognitive, and expressive needs is considered valid, and this fact is as true for the love of truth or certainty as it is in the interest of safety [28]. According to the participant’s statements in the current study, one of the categories that lead to the safe acceptance of patients is the sense of safety and security. Many qualitative and quantitative studies have also been conducted in this regard. For example, Medhekar et al. study (2019) revealed that a cause of medical tourists traveling to India for treatment is due to the concept of safety and security [29]. In the study of Sillero & Zabalegui (2019), patients considered the presence of a nurse as a factor in achieving peace and security [14]. In another study, nurses’ conversations with patients provided reassurance and empathy, created trust, and reduced anxiety. The essential factor in establishing patient safety was considering the functional competence of nurses [30]. Ridelberg et al. (2014) conducted a qualitative study about the security concept and extracted several categories, such as patient interaction, patient engagement, emotions, and nursing interest and knowledge [31]. Lanham et al. (2018) also extracted the concepts that create a sense of patient security. In this study, the ideas that impacted patients’ sense of security included information exchange between patients and nurses, problem-solving, relationship building, sense-making between patients and nurses, unresolved problems, and mismatches in tone [32].

Maintaining comforting communication was one of the categories extracted in the present study. Having no language knowledge, improper contact with the healthcare staff, and lack of knowledge about the treatment process were among the categories that caused patients to lose comfort and peace, become aggressive, and sometimes cry. Wensley et al. (2020) conducted a study entitled “Maximizing Comfort: How do patients describe important care?“ One of the categories that they extracted was cultural communication; According to the participants in the study, patients are relieved when their culture is known, and their beliefs and values ​​are respected. They also found that considering patients’ spiritual and religious beliefs from a cultural view prepares comfort and makes peace in them [33]. According to the results of the above study, cultural knowledge is critical in maintaining the patient’s comfort and peace. In another study, cultural familiarity was a way to keep patients calm. Recognition of cultural differences is an effective way to create appropriate communication, and a common language makes a sense of belonging. Considering food preferences and dealing with negative emotions are other ways to maintain patient peace [34]. Footman et al. (2015) conducted a qualitative study on dialysis services for tourists. They reported that patients have a high level of satisfaction and comfort. However, they also had challenges in the field of care. One of the challenges was the lack of familiarity with the language, which makes it difficult to communicate comfortably [35].

“Stress control” was one of the categories extracted in the present study. Stress is a central concept in biology and is now widely used in psychological, physiological, social, and even environmental research. However, the concept of stress is used to refer to various components of the stress system, including stressful stimuli, stressors, reactions to stress, and the effect of mutual stress [36]. In the present study, the medical tourists stated that they had a high level of stress on their first arrival at the hospital due to reasons such as lack of familiarity with the environment and language and inappropriate communication, all of which were categories that created stress in them. In a study performed by Mandal & Ghoshbar (2019) on the experiences of medical tourists, the results showed that medical tourists suffer from high-stress levels [37]. Considering the stress of patients and cultural differences, nurses in this study were attempting to control the stress of medical tourists by taking several measures, such as choosing a nurse who speaks the same language as the medical tourist, helping the medical tourist to get familiar with the environment, using non-verbal communication (smiling), etc. Khalili and Rahimi (2020) in their qualitative research extracted similar concepts, which included creating positive thinking, focusing on spirituality, and gaining support from caregivers [38]. Imanian et al. (2017) conducted a study on the lived experiences of medical tourists during their treatment in Tabriz hospitals. A theme identified was the attitude and behavior of nurses. The attitude and behavior of nurses played an important role in calming the mind and reducing stress and worries caused by the disease and the hospital environment in international patients [39].

Responding to inconsistent expectations was another category related to the safe acceptance of patients in the present study. International patients have different expectations, and identifying and responding to those expectations is one category that contributes to safe patient acceptance. Hwang et al. (2018) conducted a study on the experiences of medical tourists before, during, and after hospitalization, and found that patients initially have certain expectations that meeting creates positive or even negative images in them. Positive and correct interaction is an important category to satisfy the expectations of medical tourists [40]. Miller et al. (2019) conducted a study on the patient experiences of nurse-facilitated advance care planning in a general practice setting: a qualitative study. Six major themes relating to patient experiences were identified. One of the themes was the importance of knowing patients’ expectations and responding to them. According to patients, the identification of patients’ expectations by nurses affected the quality of nursing care [41]. Mishra & Sharma (2021) stated that medical tourists have some expectations from caregivers, and that is to bridge the empathy gap. In the above study, understanding the feelings and providing services as quickly as possible were among the most important expectations of medical tourists, which could be met by increasing the number of healthcare staff [42]. According to these studies, one of the expectations of medical tourists, which is also in line with the present study, is to provide healthcare services to medical tourists as quickly as possible.

## Limitations

This study also had limitations. The results of the present study, like any other qualitative study, may not be generalized to other communities or contexts. The Covid-19 pandemic created many challenges for the researcher. The borders were closed due to the Covid-19 pandemic. All departments of international patients exchanged to the Covid-19 department. On the other hand, the nurses were exhausted and engaged in dealing with the Covid-19 pandemic, and the hospitals did not allow research.

## Conclusion

The results of the present study showed that safe acceptance of cultural care is a key and significant factor in medical tourism. Trust building, safety, comforting communication, stress control, and responding to inconsistent expectations are important categories in realizing the safe acceptance of medical tourists. Nurses are aware of the categories that are effective in cultural care and safe acceptance of medical tourists, and they take the necessary measures to achieve safe acceptance. It is suggested to use quantitative studies to investigate the concepts extracted in this study and evaluate the current situation of medical tourism, especially in Iran.

## Data Availability

Empirical material generated and/or analyzed during the current study is not publicly available but from the corresponding author at a reasonable request.
